# The Causes and Treatment of Diarrhœa.—I

**Published:** 1908-10-24

**Authors:** 


### Medicine.

### THE CAUSES AND TREATMENT OF DIARRHŒA.—I.

Diarrhœa is not a disease but a symptom. Just as constipation, vomiting, jaundice may each be due to many different causes, so may diarrhœa. The latter may be almost the sole symptom in the case, and, on the other hand, it may be but one of many symptoms, as in a case of advanced phthisis with amyloid degeneration of the bowel.

When one reduces the causes of diarrhœa into the fewest possible groups it will be found that they can all, or very nearly all, be included under one or other of three main headings, namely: (1) drugs or irritating matters in the intestine; (2) abnormal conditions of the bowel walls; (3) nervous causes. It is clear, of course, that more than one cause may be at work in a single case; thus in typhoid fever there is often constipation until a purgative is taken; the diarrhœa produced may then be continued by the ulcerated state of the bowel wall.

1. *Diarrhœa due to drugs or to the presence of irritative matters in the intestine.*—The question of diarrhœa due to drugs can be dismissed in a few words. Some drugs act as mechanical irritants to the bowel wall, others as stimulators of the nerves which increase peristalsis, others again as attractors of watery fluid into the intestine. Two points perhaps deserve particular notice in regard to purgative drugs: first, an immense deal of harm is done by the use in doses that are too big of laxatives that might otherwise be harmless. It is almost invariably a bad thing to give a laxative or purgative in such a dose that a number of diarrhœic stools result; probably such an occurrence upon a single occasion would be followed by no ill result, but much of the constipation which afflicts humanity is due to the abuse of purgatives, that is to say to the constant repetition of a drug in such quantities as to cause diarrhœic motions instead of an ordinary single stool. Such temporary diarrhœa is bound to be followed by a reactionary constipation.

The second point upon which we would lay emphasis is in connection with calomel. The misuse of this drug is terrible. It is of the very highest value in certain cases, but it is a remedy which, instead of being kept for particular conditions, is ordered almost as indiscriminately and freely as is castor oil. It is too often forgotten that calomel is not merely a stimulator of peristalsis—it is an actual irritant to the bowel mucosa. Calomel can lead to the most virulent colitis, non-ulcerative to begin with, ulcerative later; and the kind of case in which it does so most readily is one in which a serious operation has been performed for a condition in which pyogenic organisms are concerned. After operation for pyosalpinx, for example, or suppurating ovarian cyst, calomel is sometimes ordered so freely that death is undoubtedly accelerated, and an acute or ulcerative colitis is found post-mortem. There is probably more misuse of calomel after surgical operations than under any other circumstances whatever.

There are other irritative matters in the intestine that may cause diarrhœa. These resolve themselves into those of bacterial origin and those not of bacterial origin. In either case the irritation is partly mechanical and partly chemical; the bacteria that cause diarrhœa do so, not directly, but indirectly by the production in the first instance of irritating toxins. In these cases the diarrhœa is a natural effort to get rid of the irritant. The intestinal evacuations are often preceded or attended by intense abdominal pain—colic; sometimes by flatulence; sometimes by borborygmi. There may or may not be vomiting. Individual peculiarities are met with; some persons suffer from diarrhœa on slighter provocation than others, and some develop the symptom from causes which would never produce it in other persons. Without going into bacteriological details, one may instance the following as causes of diarrhœa coming under the present main heading:—metallic poisons and irritants such as arsenic or corrosive sublimate, foreign bodies, indigestible and unsuitable articles of food, such as unripe fruit, decomposing food, and ptomaines. Infants and young children are particularly liable to diarrhœa. In the summer time this is chiefly due to bacterial contamination of milk—the summer diarrhœa and vomiting which is so common and so fatal in early life, traceable to exposure of the milk in unclean vessels to unclean air. At all times, especially amongst the poor, it may be due to the introduction of indigestible and unsuitable articles of food into the stomach and bowel, such as bits from their parents' plates; or still worse, bits of refuse from the floor. Errors of diet are still held to be a cause of rickets; errors of diet are certainly a cause of diarrhœa. It is small wonder, therefore, that rickets and diarrhœa often coincide.

Acute ptomaine poisoning is of two chief kinds—namely, (*a*) the variety in which there is little or no latent period between the ingestion of the peccant food and the onset of the symptoms; and (*b*) the type in which there is a latent period of a number of hours, varying from eight to twenty-four or more. The first type is best seen in eaters of crabs, lobsters, shellfish, or fried fish; acute abdominal pains, vomiting, urticaria, diarrhœa, and collapse may all come on within an hour of eating the fish. The other type is sometimes seen in a very serious and epidemic form—for instance, after a large public function at which numbers of persons may have partaken of the same food, and are all suddenly seized with symptoms in the middle of the night or early the following morning.

The chief reason for the absence of a latent period in the first type of case and its presence in the second, is that, in the fried fish instance, the ptomaines were already present when the food was consumed, whereas in the other cases the food seemed perfectly good, and it contained no ptomaines when it was eaten, but it was infected with bacteria which subsequently produced ptomaines within the intestines of the sufferers. Time was required for the bacteria to produce these ptomaines; hence the latent period.

